# Can transcutaneous perianal ultrasonography be the first-line diagnostic instrument for evaluating pediatric perianal fistulas?

**DOI:** 10.1093/gastro/goac071

**Published:** 2022-11-29

**Authors:** Yu-Wen Ding, Hao-Qiang Yin, Hong-Tao Liang, Jin-Gen Lu, Bo Wang, Chen Wang

**Affiliations:** Department of Proctology, Longhua Hospital affiliated to Shanghai University of TCM, Shanghai, P. R. China; Department of Ultrasonic, Longhua Hospital affiliated to Shanghai University of TCM, Shanghai, P. R. China; Department of Proctology, Longhua Hospital affiliated to Shanghai University of TCM, Shanghai, P. R. China; Department of Proctology, Longhua Hospital affiliated to Shanghai University of TCM, Shanghai, P. R. China; Shanghai Shumiao Health Cloud Co. Ltd, Shanghai, P. R. China; Department of Proctology, Longhua Hospital affiliated to Shanghai University of TCM, Shanghai, P. R. China

**Keywords:** pediatric perianal fistula, transcutaneous perianal ultrasound, surgical exploration, consistency

## Abstract

**Background:**

Pediatric perianal fistula is a common disorder. It is more difficult to detect the fistula tract and internal opening (IO) in children than in adults. This study aimed to evaluate the clinical diagnostic value of transcutaneous perianal ultrasound for children with perianal fistula.

**Methods:**

A retrospective review was conducted by analysing the preoperative transcutaneous perianal ultrasound and intraoperative exploration results of 203 consecutive patients who were <3 years old and diagnosed with perianal fistula. Analyses were conducted to evaluate the accuracy and consistency of utilizing the transcutaneous perianal ultrasound in the diagnosis of the complexity and location of the IO of perianal fistulas.

**Results:**

Compared with intraoperative exploration, the preoperative transcutaneous perianal ultrasonography has almost perfect agreement (Kappa = 0.881, *P *<* *0.001) in the diagnosis of fistula tract complexity and IO with a sensitivity of 92% and a specificity of 97%. In addition, both intraoperative exploration and transcutaneous perianal ultrasound diagnosis showed high consistency in the identification of the IO of perianal fistulas (Quadrant I Kappa = 0.831, Quadrant II Kappa = 0.773, Quadrant III Kappa = 0.735, Quadrant IV Kappa = 0.802, all *P *<* *0.01). The IOs were mainly distributed in Quadrants IV and II in both simple and complex fistulas.

**Conclusions:**

Transcutaneous perianal ultrasound, as a non-invasive and simple imaging technique, showed high accuracy in the diagnosis and identification of the fistula classification and IO location. It could be considered a first-line diagnostic instrument for evaluating perianal fistulas among children.

## Introduction

Perianal abscess (PA) and fistula-in-ano (FIA) are common acquired anorectal disorders affecting not only adults but also children, indicating the acute and chronic inflammatory phases of perianal infection [1]. It is important to differentiate between PA/FIA in children compared with adults since not only the pathogenesis but also the treatment differs between these entities [2]. The estimated incidence of PA in children has been reported to be at 0.5%–4.3% [3] and patients are generally <3 years old with a strong male predominance [4]. The management of PA/FIA in children and adults is different. Treatment for pediatric perianal fistula remains as conservative and surgical approaches. Drainage alone is a primary treatment that has been used for pediatric PA [5–7]. There is a large variation in the rate of recurrence and fistula formation after this initial management [1–3, 5]. However, ∼35%–42% of children presenting with an abscess ended up with FIA [1, 8]. If a child has failed conservative treatment or a complex fistula, surgical interventions may be needed [9, 10]. However, the recurrence rate after FIA surgery is as high as 13%–68% [1, 11].

The clinical practice guideline for the diagnosis of perianal fistulas for adults includes a physical examination, imaging and intraoperative exploration [12]. On account of perianal fistulas in children being tiny and obscure, it is difficult to detect them using physical examination or probing techniques when awake. Although imaging techniques such as magnetic resonance imaging (MRI) and computed tomography (CT) 3D reconstruction have been used as effective methods to describe the anatomy of anal fistulas [13, 14], the radiation exposure and examination coordination are not fit for children. 3D transanal ultrasonography can observe the structure of the anal canal and rectum from multiple planes and any angles, quantify the length of the sphincter, and clarify the internal opening (IO) and characteristics of the anal fistula. It provides more effective, comprehensive and intuitive imaging information for the evaluation of anal fistulas [15–18]. However, this technique is not suitable for patients <3 years old due to the diameter of the 3D ultrasound probe and the sphincter structures in children <5 years old are difficult to differentiate [17]. Therefore, transcutaneous perianal ultrasonography (TPUS) has proven to be a feasible non-invasive and simple imaging method for this population. The TPUS can clearly show the relationship of the fistula tracks to the levator floor in adults. It has also been used in the diagnosis of a low imperforate anus, perianal Crohn’s disease, as well as in the diagnosis of anal and rectovaginal fistulas [15, 19–21]. Nevertheless, the accuracy of TPUS to identify anal fistula classification and IO in a large group of young children has not been verified. To directly address this gap, the aim of this study was to investigate the accuracy of preoperative transcutaneous ultrasonic imaging as the diagnostic tool for perianal fistulas by comparing outcomes generated via the standard diagnosis intraoperative exploratory examination.

## Materials and methods

### Participants

This study has obtained ethics approval from the Longhua Hospital affiliated to Shanghai University of Traditional Chinese Medicine Ethics Committee, China (IRB NO.2020LCSY022). A retrospective review of hospital medical charts was conducted among all patients who were <3 years old with anal fistulas and admitted to Longhua Hospital (Shanghai, China) from January 2015 to December 2021. Diagnosis of the anal fistula was based on the International Classification of Diseases, Tenth Revision (ICD-10) code (i.e. ICD-10 K60.300).

The clinical records including preoperative perianal ultrasonography and intraoperative medical records for all consecutive included children were reviewed. The related information was extracted via a pre-defined data-collection form. Two authors (Y.W.D. and C.W.) independently reviewed the medical records and completed the data-collection forms. Patients diagnosed with congenital abnormalities, Crohn’s disease, trauma, or other surgical histories of the perianal area (except incision and drainage of PA and anal fistula surgery) and serious primary diseases were excluded. Patients with insufficient data were also excluded.

### Transcutaneous perianal ultrasound examination

#### Instruments and examination methods

**Figure 1.**
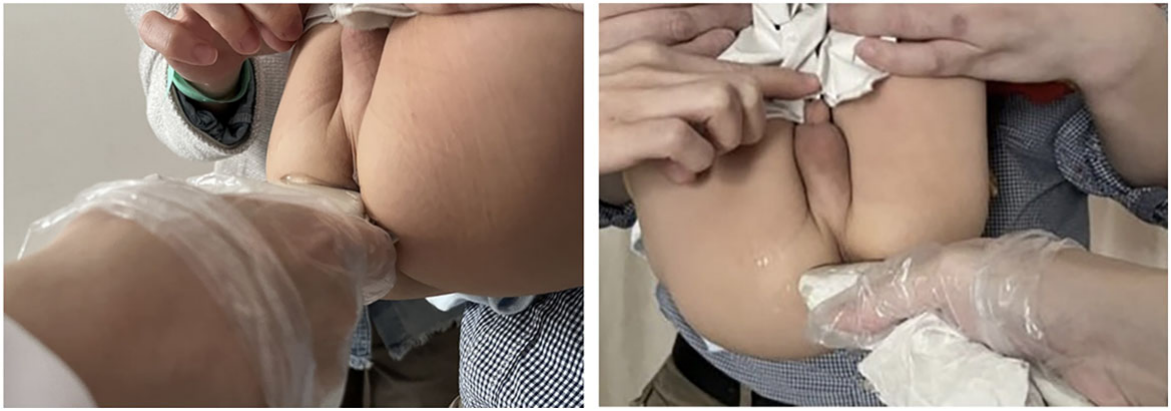
Transcutaneous perianal ultrasound examination

The ultrasound examination was performed using MyLab Twice equipment (Esaote, Genova, Italy) and a linear array probe LA435 with a frequency of 18 MHz. There is no need for patients to undertake bowel preparation and the parents were well informed to expose the child's perianal area. The assessment was carried out in the lithotomy position and the perianal scan was administered by one ultrasonic practitioner who has >20 years of experience, is familiar with the characteristics of perianal anatomy, and has experience in the diagnosis of proctological disease (Figure 1).

#### Ultrasonic diagnosis process

**Figure 2.**
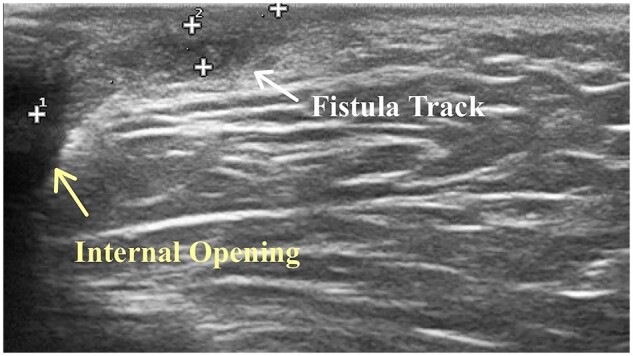
Ultrasonic manifestations of anal fistula. The yellow arrow marks the internal opening and the white arrow with “+” symbols marks the edge of the fistula track

**Figure 3.**
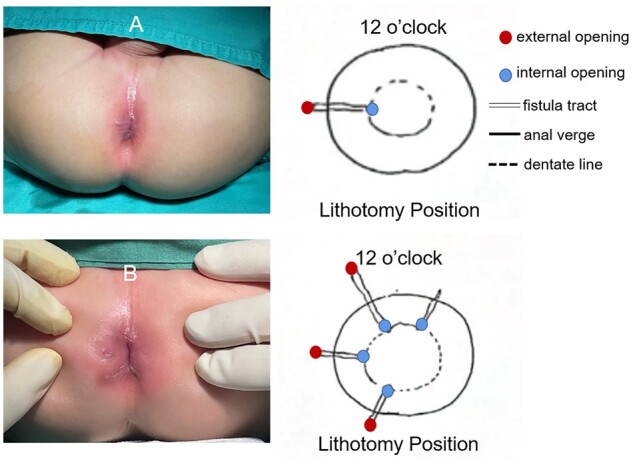
(A) The ultrasonographic record of simple anal fistulas and (B) the ultrasonographic record of complex anal fistulas

According to the ultrasound imaging manifestations of perianal fistulas, the fistula appears as a hypoechoic tract, which is followed along its crossing of the sub-epithelium, internal, or external sphincters, and through the perianal space [15, 22, 23]. The fistula tract presents as a tube-shaped anechoic or hypoechoic area and the IO can be identified after tracking the hypoechoic inward traverse of the perianal tissue. Meanwhile, the hypoechoic zone leading to the external opening on the perianal skin can be seen by tracing outward (Figure 2). During the examination, the lesion site of the fistula was carefully observed, and the fistula classification and location of the IO were recorded (Figure 3).

### Intraoperative exploration examination

After the general anesthesia, the patient was placed in the lithotomy position. A roster of three surgeons performed all operations. First, a digital examination was performed carefully to detect the fistula tract and IO. Second, surgeons confirmed the external opening to the IO by using a silver blunt-tip probe. When the external opening was temporarily closed, they used a No. 15 scalpel to make a small incision on the surface. Lastly, once the IO was detected, the probe gently came out. The fistula classification and location of the IO were then examined and recorded.

### IO distribution

**Figure 4.**
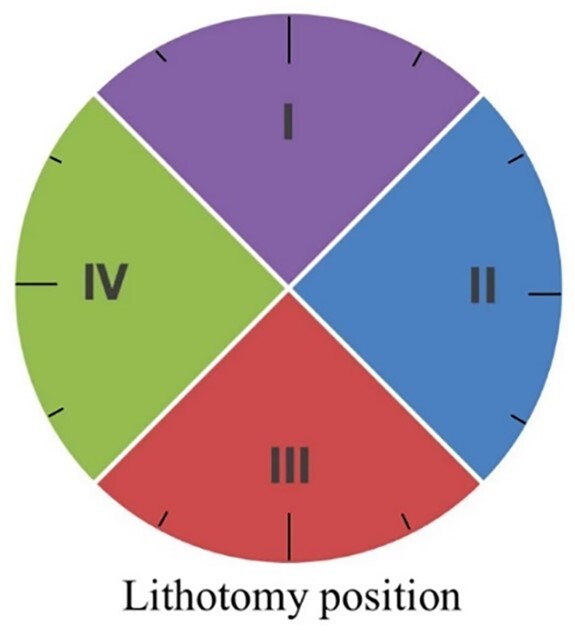
Four-quadrant distribution of the internal opening of anal fistulas. I, upper; II, left; III, lower; IV, right.

The location of each IO was mapped with the patient in lithotomy distribution of 12 points and dividing the perianal area into four quadrants (I upper, II left, III lower, and IV right; Figure 4).

### Classification of pediatric perianal fistula

Simple fistula: the perianal fistula has only one IO and one tract.

Complex fistula: the perianal fistula has either two or more IOs or branching of tracts or coexisting sepsis.

### Statistical analysis

SPSS 24 statistical software was used for statistical analysis. Continuous data were described as mean ± SD (standard deviation) and category data were expressed as number (percentage). *P *<* *0.05 was considered statistically significant.

Agreement analysis was used to test the consistency of the diagnosis of TPUS with the Cohen’s Kappa Value, which indicates the degree of consistency. Specifically, Kappa ≥ 0.80 indicates almost perfect agreement; 0.80 > Kappa ≥ 0.61 indicates substantial agreement; 0.60 > Kappa ≥ 0.41 indicates a moderate agreement; and Kappa < 0.40 indicates a poor agreement. Sensitivity and specificity were calculated to examine the accuracy of the perianal superficial ultrasound in the diagnosis of anal fistulas in infants.

## Results

### Patients’ characteristics

**Figure 5.**
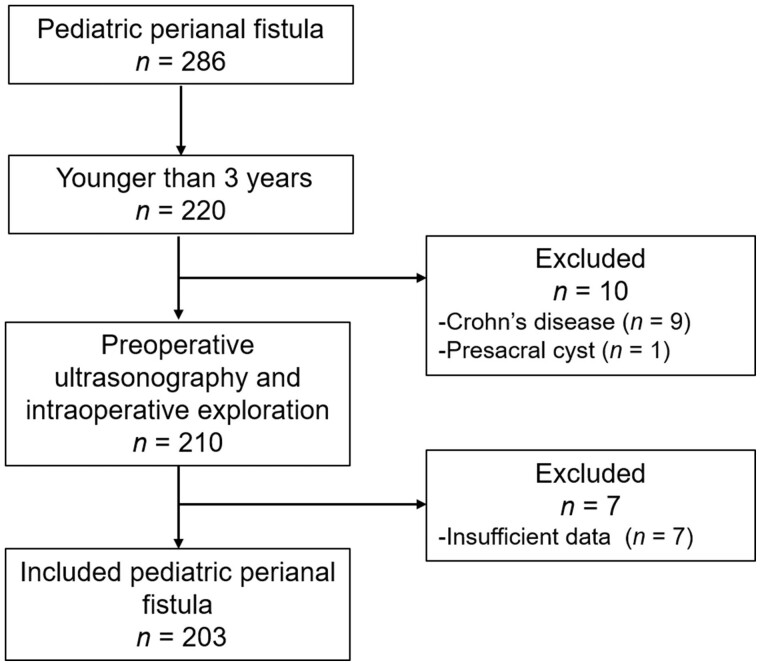
Flow chart of patients enrolled in the study

A flow chart of patients in the study is shown in Figure 5. A total of 203 medical records were thus analysed in this study. All of them were male and the mean age was 13.63 ± 8.09 months with range 3–36 months. The mean duration of the anal fistula was 9.31 ± 6.94 months. Also, 150 cases (73.89%) were drained and 5 (2.46%) had fistulotomy previously (Table 1). Eight cases (3.94%) had undergone colonoscopy, two had experienced “chronic colitis,” three had experienced “allergic colitis,” two had experienced “colonic follicular hyperplasia,” and one had experienced a “small amount of nodular lymphoid hyperplasia of terminal ileum and colon.”

**Table 1.** Baseline characteristics of the patients (*n *=* *203) in the study

Characteristics	*n* (%)
Age, months	
<12	118 (58.13)
12–36	85 (41.87)
Previous incision and drainage, *n*	
0	53 (26.11)
1	97 (47.78)
2	33 (16.26)
3	11 (5.42)
4	8 (3.94)
5	1 (0.49)
Previous fistulotomy history	
No	198 (97.54)
Yes	5 (2.46)

### Ultrasonic examination

**Figure 6.**
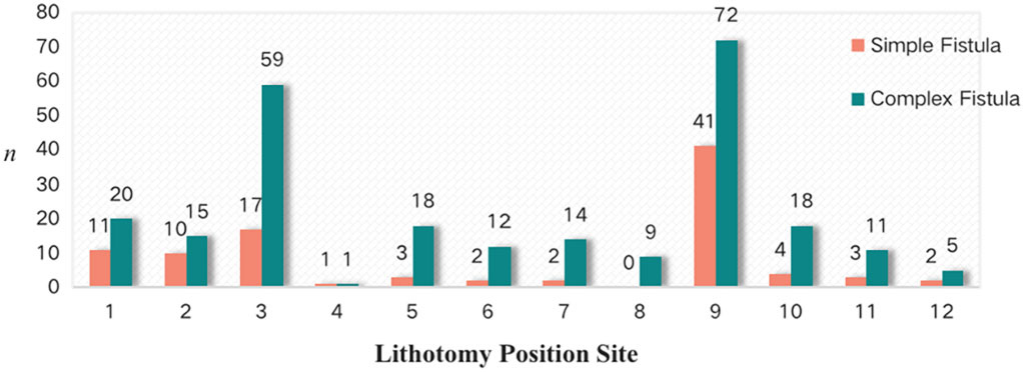
The locations of internal openings (IOs) via transcutaneous perianal ultrasound examination. *n* refers to the number of IOs.

**Figure 7.**
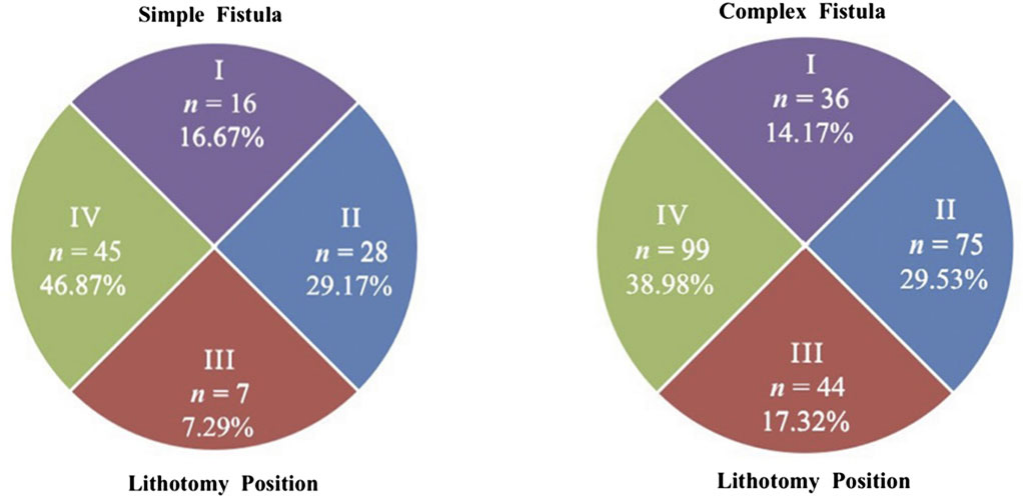
The distribution of internal opening (IO) locations in quadrants via transcutaneous perianal ultrasound. *n* refers to the number of IOs.

There were 96 (47.29%) cases diagnosed with a simple anal fistula via the transcutaneous perianal ultrasound examination, manifesting as an oblique tubular hypoecho passing through the perianal tissue and connecting to the anal canal with a single IO. The remaining 107 (52.71%) cases were diagnosed with a complex anal fistula, which had either two or more IOs or two or more tubular hypoechoes passing through the perianal tissues around the anus. A total of 254 IOs were detected among those with complex anal fistulas, with a mean of 2.37 ± 0.77 IOs per patient. The sites of the IOs via perianal ultrasound are shown in Figure 6. The distribution of the IO locations of the simple and complex anal fistulas is monitored into quadrants separately in Figure 7.

### Surgical exploration examination

**Figure 8.**
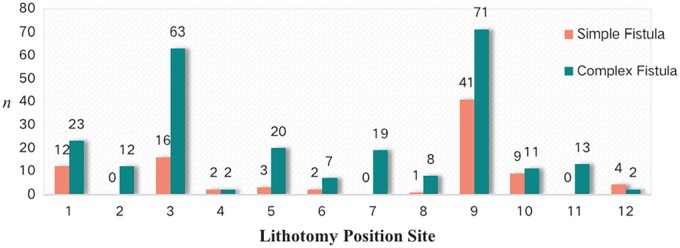
The location of internal openings (IOs) via surgical exploration. *n* refers to the number of IOs.

**Figure 9.**
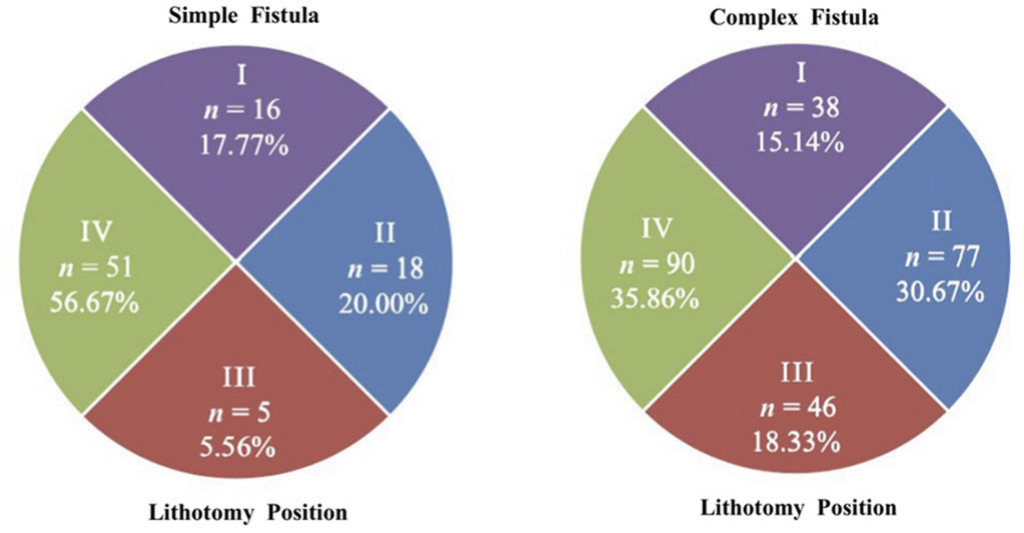
The distribution of internal opening (IO) locations in quadrants during surgery. *n* refers to the number of IOs.

According to the intraoperative exploration examination, 90 cases (44.33%) of simple anal fistulas were found with a single IO and 113 cases (55.66%) were diagnosed with complex anal fistulas with 251 IOs. That is, each patient with a complex anal fistula had an average of 2.22 ± 0.70 IOs. The sites of the IOs during surgical exploration are shown in Figure 8. The distribution of the IO locations of the simple and complex anal fistulas is monitored into quadrants separately in Figure 9.

### Agreement analysis

#### Complexity of anal fistulas

TPUS and intraoperative exploration were almost perfectly consistent in the classification of simple and complex perianal fistulas in children (Kappa = 0.881, *P < *0.001) (Table 2). The ultrasound method has a sensitivity of 92% and a specificity of 97% for complex anal fistula diagnosis.

**Table 2.** Consistency test results of ultrasonography and intraoperative exploration for the classification of pediatric perianal fistulas

Perianal ultrasonography	Intraoperative exploration		Total
	Simple fistula	Complex fistula	
Simple fistula	87	9	96
Complex fistula	3	104	107
Total	90	113	203

Kappa value = 0.881, *P < *0.001; sensitivity = 92%, specificity = 97%.

#### IO distribution

The transcutaneous perianal ultrasound and intraoperative exploration are almost perfectly consistent in determining the distribution of the IOs in Quadrants I and IV (Kappa > 0.80, *P *<* *0.01) and were substantially consistent in Quadrants II and III (Kappa > 0.61, *P *<* *0.01; Table 3).

**Table 3.** Agreement analysis of perianal ultrasound and intraoperative exploration in the IO distribution of pediatric perianal fistulas

IO distribution	Perianal ultrasound	Intraoperative exploration		Kappa value	*P*-value
		Yes	No		
Quadrant I	Yes	40	5	0.831	<0.01
	No	7	151		
Quadrant II	Yes	87	15	0.773	<0.01
	No	8	93		
Quadrant III	Yes	37	12	0.735	<0.01
	No	7	147		
Quadrant IV	Yes	125	10	0.802	<0.01
	No	8	60		

## Discussion

The management of perianal fistulas in children remains empiric and comprises conservative (with or without antibiotics), as well as surgical approaches (drainage, fistulotomy, fistulectomy, and seton placement) [2]. Conservative and non-operative strategies should be the first-line treatment in this specific age group, whereas surgery should preferentially be reserved for patients with failed conservative management or complex fistulas [1]. Preoperative precise imaging of complex fistulas can reduce the recurrence rate. This is the first study to focus on the accuracy of transcutaneous ultrasonic examination for pediatric perianal fistulas including a comparison with surgical exploration. Among the 203 consecutive children's medical records reviewed, 52.71% and 55.66% were diagnosed with complex anal fistulas (complexity relates to the number of IOs or the branching of tracks and coexisting sepsis) via ultrasonography and intraoperative exploration, respectively. This result showed “almost perfect” consistency. Most patients with complex anal fistulas have two or more IOs and one toddler (19 months old with an 18-month disease course, Figure 3B) had four IOs.

It is worth noting that 12 cases in this study were found to be inconsistent in the classification of an anal fistula between the ultrasonography and intraoperative exploration examinations. To be specific, three cases were identified as complex anal fistulas via ultrasound examination but simple anal fistulas via intraoperative exploration. Such a finding may be related to the fact that ultrasound examination is confused with the scar of previous drainage or fistulotomy [24]. Regarding the other nine cases, ultrasound examination suggested simple fistulas while intraoperative exploration indicated complex anal fistulas accompanied by small branches or coexisting sepsis. For seven of these nine patients, there was a long interval between the ultrasound examination and surgical exploration, and the differences in the results between the two methods may be due to changes in the progression of the perianal fistulas.

In terms of adults, based on Goodsall’s rule, IOs can be estimated from 70% of anal fistulas [25]. However, due to the different pathogenesis of perianal fistulas and the anatomy of the anal sphincters between children and adults, IO identification among children does not meet Goodsall’s rule [26–28]. The results of this study confirmed that the distribution of the IOs of perianal fistulas in children is different from that in adults.

Meanwhile, most anal fistulas in children are superficial, radial, and straight without involving the anal sphincter and levator muscle, so it makes sense that surgeons can perform fistulotomy even for complex anal fistulas in children without a matter of concern about incontinence. Transcutaneous ultrasonography examination suggested a good consistency with intraoperative exploration for IO identification. Both ultrasonography and intraoperative exploration examinations confirmed that the IOs of simple anal fistula cases was mostly located at lithotomy 9 o’clock, while the IOs of the complex anal fistula cases were mostly located at lithotomy 3 and 9 o’clock. By further dividing the perianal area into quadrants, the IOs of anal fistulas can be clearly found mainly concentrated in Quadrants IV and II. Only eight cases (3.94%) of complex fistulas had a cross-quadrant distribution of fistulas and only one case (5 months old, with 2-month course of disease) had a horseshoe fistula.

The currently preferred method for assessing structural integrity is endosonography morphology [17, 19, 29]. The ultrasonographer can follow a fistula longitudinally along its course, which may lie outside of the focal distance of an endoanal probe for larger children and adults. However, the assessment of the structures in children <5 years of age is difficult to detect. As presented by this study, transcutaneous ultrasonography could be used as a supplementary technique for patients under 3 years old, and shows high accuracy and sensitivity in fistula detection. This technique requires a substantial learning curve. An experienced ultrasound practitioner is required to be familiar with the characteristics of perianal anatomy and have experience in proctological disease. Good exposure of the patient’s perianal area and suitable ultrasonic equipment with high frequency and a small probe are important for the diagnosis of pediatric perianal fistulas.

There are two limitations to the interpretation of the findings of this study. First, the study is limited by its retrospective design. Second, there was no blinded comparison between reviewers and therefore no inter-observer and intra-observer variability. However, further investigation is warranted to compare children with different age distributions and the dimensional description of fistulas with blinded comparison.

## Conclusions

TPUS has shown high accuracy and consistency compared with surgical exploration with regard to the diagnosis of perianal fistulas in children. Such an ultrasonography examination can clearly display the fistula classification and the location of the IOs, with the advantages of convenient operation and being non-invasive and simple. It will play a significant role in supporting clinical evaluation and surgical intervention in children with perianal fistulas. Further prospective research is needed to explore the characteristic of pediatric perianal fistulas.

## Authors’ Contributions

The study conception, design, and manuscript writing were done by C.W. Material preparation, data collection, and analysis were performed by Y.W.D., H.Q.Y., H.T.L., and J.G.L. Data analysis was done by B.W. All authors read and approved the final version of the manuscript.
